# Antibody Response to Paramyxoviruses in Paget’s Disease of Bone

**DOI:** 10.1007/s00223-017-0265-4

**Published:** 2017-03-31

**Authors:** Micaela Rios Visconti, Ricardo Usategui-Martín, Stuart H. Ralston

**Affiliations:** The Centre for Genomic and Experimental Medicine, MRC Institute of Genetics and Molecular Medicine, Western General Hospital, University of Edinburgh, Edinburgh, EH4 2XU UK

**Keywords:** Paget’s disease of bone, Paramyxovirus, Measles, Distemper, Genetic

## Abstract

Paget’s disease of bone (PDB) is a common skeletal disorder characterised by focal abnormalities of increased and disorganised bone turnover. Genetic factors play a central role in the pathogenesis of PDB but environmental factors also contribute. Measles virus (MV), respiratory syncytial virus (RSV) and canine distemper virus (CDV) have all been implicated as potential disease triggers but the data are conflicting. Since chronic paramyxovirus infection with measles is known to be accompanied by increased production of antiviral antibodies, we have analysed circulating concentrations of antibodies to MV, CDV, and RSV as well as mumps, rubella and varicella zoster virus (VZV) in 463 patients with PDB and 220 aged and gender-matched controls. We also studied the relation between viral antibody concentrations and various markers of disease severity and extent in 460 PDB patients. A high proportion of cases and controls tested positive for antiviral antibodies but there was no significant difference in circulating antibody concentrations between PDB cases and controls for MV, CDV, RSV, rubella or VZV. However, mumps virus antibody levels were significantly higher in the PDB cases (mean ± SD = 3.1 ± 0.84 vs. 2.62 ± 0.86. *p* < 0.001). There was no association between disease severity and circulating antibody concentrations to any of the viruses. In conclusion, we found no evidence to suggest that PDB is associated with abnormalities of immune response to measles or other paramyxoviruses, although there was evidence of a greater antibody response to mumps. The results do not support that hypothesis that PDB is associated with a persistent infection with measles or other paramyxoviruses.

## Introduction

Paget’s disease of bone is a common skeletal disorder characterised by focal abnormalities of increased and disorganised bone turnover at one or more skeletal sites [1]. Many patients are asymptomatic but those that do come to clinical attention can develop various complications including bone pain, deformity, pathological fractures, osteoarthritis, deafness and rarely osteosarcoma [2, 3]. Paget’s disease is a complex disorder. Current evidence suggests that genetic factors play a key role in susceptibility [4], but there is also a strong environmental component evidenced by the fact that the disease has become less common and less severe in several countries over recent decades [5–7]. Several potential environmental triggers for Paget’s disease of bone (PDB) have been suggested including dietary calcium intake [8], vitamin D deficiency [9] and excessive biomechanical loading of affected bones [10], but the most widely studied is paramyxovirus infection [11, 12]. Paramyxoviruses were first implicated as a potential trigger of PDB by the morphological finding of nuclear inclusion bodies in osteoclast nuclei which were thought to resemble measles nucleocapsids [13, 14]. Despite extensive research over the past 30 years, the role of paramyxovirus infection in the pathogenesis of PDB remains controversial [15, 16]. Some researchers have reported finding evidence of measles virus (MV) nucleic acids or antigens in cells and tissues from patients with the disease [17–20]. Respiratory syncytial virus antigens were also detected in one study [21], and in another evidence was presented to suggest that antigens for both MV and respiratory syncytial virus (RSV) may be present [22]. Canine distemper virus (CDV) nucleic acids have also been reported by one group to be present in Pagetic bone cells [23, 24], whereas other researchers failed to find evidence of paramyxovirus nucleic acids or protein in bone tissue or peripheral blood from PDB patients [15, 25–29]. A characteristic feature of the persistent measles infection, subacute sclerosing panencephalitis, is the presence of a marked increase in antiviral antibodies both in the serum and cerebrospinal fluid [30, 31]. Based on this observation, several researchers have measured antibodies to paramyxoviruses in patients with PDB [32–35]. These studies yielded negative results, but the sample sizes were small and had limited power to detect possible differences between PDB cases and controls. Here, we have evaluated the antibody response to paramyxoviruses in PDB in the largest study performed to date using a case control design and determined if antibody concentrations were associated with disease extent or complications. We also evaluated the antibody response to other common viral infections not previously implicated in the pathogenesis of PDB including mumps, rubella and varicella zoster virus.

## Patients and Methods

### Patients

The study cohort comprised 463 patients who participated in the Paget’s disease, Randomised Trial of Intensive versus Symptomatic Management (PRISM) study (ISRCTN12989577) [36, 37]. Participants were included in this study if sufficient serum was available from the baseline study visit for analysis of the antibodies of interest. One subject included in this study had a history of osteosarcoma. The controls comprised 202 age and gender match subjects not known to have PDB who were predominantly spouses of PRISM participants.

### Clinical Assessments

The clinical assessments performed within the PRISM study have been previously described [36]. Health-related quality of life was assessed by the SF-36 questionnaire. Deformity was assessed by the attending physicians who were asked to assess whether the patient had clinical evidence of bone deformity using a three-point scale as follow: 0 = no deformity; 1 = mild or moderate deformity and 2 = severe deformity. The presence of bone pain was recorded and physicians were asked to assess if they thought the pain was caused by PDB. Information was collected on previous fractures and whether they had occurred in affected bone; on orthopaedic surgical procedures; on the use of a hearing aid for deafness in those with skull disease; on age at first diagnosis of PDB; and family history of PDB. The extent of PDB was recorded based on involvement on bone scan examination. Information was recorded on previous bisphosphonate treatment, and the number of treatment courses given. Based on the clinical data, we devised a composite scoring system for disease severity as previously described [38] taking into account the number of affected bones, age at first diagnosis, family history, presence of bone pain, pathological fracture, orthopaedic surgery for PDB, bone deformity, deafness associated with skull involvement and previous bisphosphonate treatment for PDB.

### Genotyping

Genotyping was conducted for *SQSTM1* mutations by DNA sequencing on DNA extracted from peripheral blood using standard techniques as previously described [38].

### Detection of Antiviral Antibodies

Enzyme-linked immunosorbent assays (ELISA) were used for the detection and quantitative determination of IgG antibodies to MV, Rubella, Mumps, Varicella Zoster Virus (VZV), RSV and CDV. The ELISA were performed on serum samples that had been collected at the baseline visit of the PRISM study between 2001 and 2004 and stored frozen at −80^o^c until analysis in the present study. We used the Trinity Biotech Captia assays ^TM^ for MV (catalogue number 2326000), mumps (catalogue number 2325900) rubella (catalogue number 2325300) and VZV (catalogue number 2325600). We used the MP Biomedical IgG ELISA (catalogue number 071-516002) for RSV. For CDV, we used the ImmunoComb^®^ antibody test kit. All assays were performed according to the manufacturer’s instructions. The results of the ELISA assays were read on a Synergy HT Multi-Mode Micro plate reader (Bio-Tek), 450 nm filter. Dual wavelength was used and the reference filter set to 600–650 nm. All samples were tested in duplicate and the mean value of the two samples calculated. Duplicate samples that yielded values that differed by more than 20% were repeated. With the exception of CDV, data were expressed as the immune status ratio (ISR). This was calculated by dividing the optical density value of the patient sample by a calibrator value provided with the kit. These results were converted to International Units (IU) using the natural exponential function *f*(*x*) = e^*x*^. For CDV, semi-quantitative analysis was performed based on a colour scale provided with the kit using Comb Scan software. The reference ranges are shown in Table 1.

**Table 1:** Reference ranges for viral antibodies

Virus	Negative	Indeterminate	Positive
Measles	≤0.06	0.061–0.09	≥0.091
Mumps	≤0.9	0.91–1.09	≥1.10
Rubella	≤6.5	6.6–8.1	≥8.2
Varicella zoster	≤0.11	0.12–0.14	≥0.15
Respiratory syncytial virus	≤0.54	0.55–1.09	≥1.10
Canine distemper	0	1–2	≥3

Values are in international units with the exception of CDV which are arbitrary units. Negative results indicate that there is no serological evidence of previous infection; positive results indicate a previous infection; values in between are indeterminate

### Statistical Analysis

Differences in viral antibody concentrations in PDB cases and controls were assessed quantitatively using Student’s *t*-test and semi-quantitatively by χ^2^ test in which patients were categorised into two groups if antibody concentrations suggested there had been previous infection. The relation between viral antibody titres and disease extent was evaluated by dividing PDB subjects into three groups based on the severity score and comparing viral antibody titres and other clinical characteristics between the groups by ANOVA or χ^2^ test as appropriate. The significance level was set at 0.008 to account for the fact that antibodies against six different viruses were tested.

## Results

### Antiviral Antibodies in PDB Cases and Controls

Demographic characteristics and antiviral antibody concentrations in PDB cases and controls are shown in Table 2. A high proportion of PDB cases and controls tested positive for measles virus, rubella, VZV, mumps and RSV indicating previous infection, with no significant difference between the groups. All cases and controls tested positive for at least one virus and more than 90% tested positive for four of the viruses. Antibodies were also detected that cross-reacted with distemper virus in about 45% of PDB cases and controls with no difference between the groups. Quantitative analysis showed that antibody concentrations did not differ between cases and controls for MV, CDV, RSV and VZV (Table 2). However, antibody concentrations for mumps virus were significantly higher in PDB cases as compared with controls (3.00 ± 0.85 vs. 2.56 ± 0.89; *p* < 0.001). For rubella virus, antibody concentrations in PDB cases were also higher, but this was not significant taking multiple testing into account (29.1 ± 11.4 vs. 27.30 ± 9.82, *p* = 0.039).

**Table 2:** Clinical characteristics and antibody concentrations in PDB cases and controls

	PDB (*n* = 463)	Controls (*n* = 221)	*p-*value
Age (years)	72.4 ± 8.0	72.2 ± 8.4	0.75
Male	240 (51.8%)	114 (51.5%)	0.95
Age at diagnosis	64.3 ± 10.5	–	
Family history PDB	70 (15.1%)	–	
*SQSTM1* mutation	38 (8.4%)		
Number of affected sites		–	
1	222 (47.1%)		
2	141 (30.4%)		
3	62 (13.4%)		
>4	38 (8.2%)		
Fractures in pagetic bone	47 (10.1%)		
Orthopaedic surgery	83 (17.9%)		
Previous bisphosphonate	353 (76.2%)	–	
Deafness and skull involvement	34 (7.3%)		
Bone deformity	172 (37.1%)		
Bone pain	345 (74.5%)		
Adjusted alkaline phosphatase	1.18 ± 1.01	–	
Measles virus			
Antibody concentration (IU/ml)	3.00 ± 12.3	2.32 ± 6.16	0.36
Previous infection	459 (99.1%)	217 (98.2%)	0.28
Respiratory syncytial virus			
Antibody concentration (IU/ml)	227 ± 144	220 ± 143	0.57
Positive serology	425 (91.8%)	204 (92.3%)	0.881
Canine distemper virus			
Antibody concentration (IU/ml)	2.37 ± 1.08	2.39 ± 1.16	0.79
Positive serology	208 (45.1%)	100 (45.2%)	1.00
Mumps virus			
Antibody concentration (IU/ml)	3.00 ± 0.85	2.56 ± 0.89	0.0001
Positive serology	451 (97.4%)	210 (95.0%)	0.11
Varicella zoster virus			
Antibody concentration (IU/ml)	0.872 ± 0.728	0.784 ± 0.741	0.147
Positive serology	448 (96.8%)	215 (97.3%)	0.81
Rubella virus			
Antibody concentration (IU/ml)	29.1 ± 11.4	27.30 ± 9.82	0.039
Positive serology	451 (97.4%)	215 (97.3%)	1.00

The values shown are mean SD and number (%). The *p*-values refer to differences between cases and controls assessed by Students *t*-test, Fishers exact test or χ^2^ test. For MV, RSV, Mumps, VZV and Rubella participants were coded as having positive serology when antibody concentrations were above the reference range suggested by the manufacturer as indicating previous infection. Information on antibody concentrations for CDV was missing for two PDB patients. For CDV, subjects who tested positive or high were considered to have positive serology

### Relation Between Severity of PDB and Antiviral Antibodies

To determine if circulating antibody concentrations were related to the severity of PDB, patients were grouped into three categories based on the disease severity score as previously described [38]. Mild disease was defined as a score of 3 or less, moderate disease as a score of 4 to 6 and severe disease as a score of 7 or more. The result is shown in Table 3. There was no association between disease severity and circulating antibody concentrations to any of the viruses tested. We repeated the analysis for number of affected bones but no association was observed (data not shown). The presence of *SQSTM1* mutations was associated with greater severity. Not surprisingly, patients with more severe disease had a significantly lower physical functioning as assessed by the SF36 instrument. Interestingly, there was difference between the severity groups in SF36 bodily pain or mental functioning. The patient with a history of osteosarcoma tested positive for all the viruses studied, except CDV where the results were negative. The circulating antibody concentrations were unremarkable in this patient.

**Table 3:** Antiviral antibodies and other variables in relation to severity of PDB

	Mild PDB (*n* = 152)	Moderate PDB (*n* = 163)	Severe PDB (*n* = 145)	*p*-value
Age (years)	74.7 ± 6.2	71.2 ± 8.6	71.5 ± 8.6	<0.0001
Male gender	85 (55.9%)	78 (47.8%)	76 (52.4%)	0.35
Measles virus (IU/ml)	4.5 ± 18.6	1.9 ± 6.0	2.4 ± 8.9	0.13
Respiratory syncytial virus (IU/ml)	224.5 ± 147.9	228.8 ± 144.7	227.5 ± 141.6	0.96
Canine distemper virus (IU/ml)	2.40 ± 1.11	2.42 ± 1.11	2.24 ± 1.01	0.30
Mumps virus (UI/ml)	2.96 ± 0.91	3.05 ± 0.82	3.0 ± 0.81	0.60
Varicella zoster virus (IU/ml)	0.86 ± 0.76	0.88 ± 0.66	0.88 ± 0.77	0.95
Rubella virus (IU/ml)	28.6 ± 11.6	30.0 ± 12.0	28.9 ± 10.7	0.38
*SQSTM1* mutation	3 (2.0%)	16 (9.8%)	20 (13.8%)	0.001
SF36 bodily pain	40.2 ± 10.9	38.6 ± 10.7	38.0 ± 10.5	0.19
SF36 physical functioning	38.1 ± 11.0	36.2 ± 11.5	34.3 ± 10.4	0.02
SF36 mental functioning	47.9 ± 11.0	49.6 ± 11.3	47.7 ± 11.8	0.31

Patients with mild PDB had a disease severity score of 3 or less, those with moderate a score of 4–6 and those with severe PDB a score of 7 or more. Values are mean ± standard deviation or numbers (%). The *p*-values refer to differences across groups, as assessed by ANOVA

### Antiviral Antibodies and *SQSTM1* Mutations

It has previously been suggested that measles virus infection may interact with *SQSTM1 *mutations to act as a trigger for PDB [39]. To determine whether antibody concentrations were associated with *SQSTM1* mutation status, we performed a subgroup analysis in which we analysed circulating antibody concentrations in *SQSTM1* mutation positive and negative PDB cases as compared with controls. The results are summarised in Table 4. There was no difference in circulating antibody concentration to any of the viruses tested according to *SQSTM1* mutation status, although concentration of mumps virus antibodies were higher in *SQSTM1* negative and *SQSTM1* positive PDB cases as compared with *SQSTM1* negative controls.

**Table 4:** Antiviral antibodies in PDB cases and controls according to *SQSTM1* status

	PDB *SQSTM1* +ve (*n* = 38)	PDB *SQSTM1* −ve (*n* = 425)	Control *SQSTM1* −ve (*n* = 221)	*p*-value
Age (years)	71.1 ± 6.1	72.6 ± 8.0	72.2 ± 8.6	0.56
Male gender	17 (44.7%)	223 (52.5%)	114 (51.6%)	0.65
Measles virus				
Antibody concentration (IU/ml)	2.65 ± 8.8	2.98 ± 12.5	2.31 ± 6.2	0.75
Previous infection	38 (100%)	421 (99.1%)	217 (98.2%)	0.49
Respiratory syncytial virus				
Antibody concentration (IU/ml)	236.5 ± 138.9	225.9 ± 144.7	220.3 ± 143.4	0.78
Previous infection	35 (92.1%)	390 (91.8%)	204 (92.3%)	0.97
Canine distemper virus				
Antibody concentration (IU/ml)	2.31 ± 1.35	2.37 ± 1.05	2.39 ± 1.16	0.91
Previous infection	14 (37.8%)	194 (45.7%)	100 (45.2%)	0.64
Mumps virus				
Antibody concentration (IU/ml)	3.00 ± 0.92	3.00 ± 0.84	2.56 ± 0.89	0.0001
Previous infection	37 (97.4%)	414 (97.4%)	210 (95.0%)	0.29
Varicella zoster virus				
Antibody concentration (IU/ml)	0.86 ± 0.72	0.87 ± 0.73	0.78 ± 0.74	0.34
Previous infection	36 (94.7%)	412 (96.9%)	215 (97.3%)	0.73
Rubella virus				
Antibody concentration (IU/ml)	30.8 ± 11.9	28.9 ± 11.4	27.3 ± 9.8	0.09
Previous infection	38 (100%)	413 (97.2%)	217 (97.3%)	0.35

Values are mean ± SD or number (%). The *p*-values are from ANOVA for antibody concentrations and from χ^2^ test for the proportion of patients with previous infection. Information on antibody concentrations for CDV was missing for two PDB patients; one in the *SQSTM1* positive and one in the *SQSTM1* negative group

## Discussion

The aim of this study was to determine if antibody response to measles virus, RSV or distemper virus was associated with the occurrence or severity of PDB. In addition to testing for antibodies to the paramyxoviruses which have previously been implicated in PDB [11, 12, 22], we also measured antibody concentrations to other common viruses such as mumps, rubella and varicella zoster virus.

The results showed that a high proportion of patients in both the PDB group and the control group had circulating antibodies to the viruses tested indicating that previous infection had occurred. For most viruses, the prevalence of those that tested positive was greater than 95% with no difference between cases and controls. These results confirm the findings of previous small scale studies which failed to find a difference in antiviral antibody concentrations between PDB cases and controls [32–35]. For CDV, only about 45% of cases and controls tested positive. Since there is extensive homology within the paramyxovirus family [40–44], it is unclear if these antibodies truly represented previous distemper virus infection or were cross reacting antibodies due to exposure to other paramyxoviruses such as measles [45]. Further studies will be required to investigate this fully. On quantitative testing, there was no significant difference in circulating antibody concentrations between cases and controls except for mumps virus where concentrations were significantly higher in cases. This raises the possibility that the immune response to mumps may be altered in PDB and suggests that the role of mumps in PDB might be worth further study.

We also sought to determine if the levels of virus antibody were associated with the severity of PDB. For this analysis, patients were divided into three categories of mild moderate and severe PDB based on disease extent and complications as previously described [38]. No significant association between circulating antibody levels and disease severity or extent was observed. Given that persistent virus infections such as SSPE are associated with high titres of antibody production [30, 31], this observation argues strongly against the possibility that there is a state of persistent paramyxovirus viral infection in PDB contrary to what has been suggested [46]. While the present study provides no support for the hypothesis that patients with PDB have persistent paramyxovirus infection, the study design cannot completely rule out the possibility that previous exposure to a viral illness early in life might play a role in PDB. However, as previously reported, there was a significant association between disease severity and the presence of *SQSTM1* mutations [38, 47] emphasising the importance of genetic factors in the pathogenesis of PDB and its severity. In summary, the present study does not provide support for the notion that persistent paramyxovirus infection is involved in the pathogenesis of PDB.
